# Associations between the home environment and childhood weight change: a cross-lagged panel analysis

**DOI:** 10.1038/s41366-022-01170-8

**Published:** 2022-06-24

**Authors:** Alice R. Kininmonth, Stephanie Schrempft, Andrea Smith, Louise Dye, Clare Lawton, Abigail Fisher, Clare H. Llewellyn, Alison Fildes

**Affiliations:** 1 School of Psychology, University of Leeds, Leeds, LS2 9JT UK; 2 Research Department of Behavioural Science and Health, University College London, London, UK; 3 Unit of Population Epidemiology, Division of Primary Care, Geneva University Hospitals, Geneva, Switzerland; 4 MRC Epidemiology Unit, University of Cambridge, Cambridge, UK

**Keywords:** Risk factors, Epidemiology

## Abstract

**Background:**

The obesogenic quality of the home environment is hypothesised to play an important role in children’s weight development but few prospective studies have investigated relationships between the home environment and adiposity across childhood.

**Objective:**

To investigate the continuity and stability of the home environment from ages 4 to 12, and bi-directional relationships between the home environment and BMI-SDS from ages 4 to 12.

**Methods:**

Parents from the Gemini cohort completed the Home Environment Interview (HEI), a comprehensive measure of the obesogenic home environment, when their children were aged 4 and 12 (*n* = 149 families, *n* = 298 children). The obesogenic home environment was measured using four composite scores capturing the food, activity, media environments, and the overall home environment. Child weights and heights were used to calculate BMI-SDS. Continuity was assessed with Pearson’s correlations between scores at each time point, and stability by changes in mean scores over time. Cross-lagged analyses were performed (HEI composites at age 4 to BMI-SDS at age 12 and the reverse) to measure the magnitude and direction of associations.

**Results:**

The home environment showed moderate-to-high continuity from ages 4 to 12 (*r* = 0.30–0.64). The overall home environment *(r* *=* 0.21, *p* < 0.01) and media composites *(r* = 0.23, *p* < 0.01) were cross-sectionally associated with child BMI-SDS at age 12, but not at age 4. Longitudinally, the home media environment at age 4 predicted increases in child BMI-SDS at age 12 (*β*; 95% CI = 0.18; 0.08,0.28, *p* < 0.01). No associations were observed for the reverse path, or the remaining composites (the overall, food and activity) in either direction.

**Conclusion:**

This study provides evidence that the obesogenic home environment tracks across childhood and highlights the importance of the early home media environment for child weight development. The findings provide insight into key aspects of the home environment that could be targeted when developing obesity treatment or prevention strategies.

## Introduction

A growing body of evidence suggests early childhood experiences are important for predicting obesity risk [1–3]. The family home environment is thought to be particularly influential in shaping early life obesogenic dietary and physical activity behaviours associated with excess weight gain [4–7]. However, few studies have successfully demonstrated robust relationships between measures of the home environment and weight development in childhood [8].

The ‘obesogenic’ home environment can be conceptualised in terms of three separate domains: the food, physical activity and media domains. Each domain consists of physical (e.g. availability and access) and social factors (e.g. caregiver modelling, rules and limit setting), which have been shown to predict a child’s dietary intake and activity levels [9–11], and thus deemed important for weight trajectories.

Consistent evidence for the role of the home environment in childhood weight development has not yet been established. For the home media domain, reliable cross-sectional associations have been observed between greater availability of, and access to, electronic devices and higher adiposity outcomes in children aged 3–12 years old [8, 12–16]. Evidence for the role of the home food domain is more mixed. Some studies have demonstrated cross-sectional associations between greater availability and access to energy-dense foods and beverages with higher BMI in pre-school [17] and school-aged children [18], while other studies report no association [9, 19–21]. Findings for associations between the home physical activity domain and child weight are also equivocal. Studies have reported access to physical activity equipment and garden space at home were associated with lower BMI z-scores [22, 23], but others have reported inverse [24] or null associations [25, 26]. Findings are similarly mixed for social aspects of the home environment, such as parental modelling, and parental rules and limit setting [8]. This conflicting evidence likely reflects the fact that individual aspects of the home environment alone have limited influence on child weight development [8, 11]. Composite measures that take into account multiple aspects of the home environment are required to evaluate the obesogenic risk within the home with greater precision, and to explore relationships with child weight trajectories.

Longitudinal research in this area is limited and has tended to focus on a single aspect of the home environment [8], with mixed results. One large longitudinal study of UK children (*n* = 12,556) found that having a TV in the child’s bedroom at age 7 was associated with greater risk of overweight at age 11 [27]. However, a prospective Australian study revealed no association between home food availability and child BMI z-scores in 5–6-year-old (*n* = 161) and 10–12-year-old (*n* = 132) children [20]. Longitudinal research has also been largely unidirectional, measuring the home environment at a single time point and examining the influence on child weight in later life, rather than the reverse. Gaining insights on directionality of associations is important as it allows us to understand whether the home environment is driving child weight or child weight is driving the obesogenic nature of the home environment. Two cross-sectional studies of a sample of British children in early (4 years) and later (12 years) childhood used a comprehensive measure of obesogenic risk within the home food, physical activity, and media environments and revealed that children living in higher-risk home environments had poorer diets, engaged in less physical activity and more sedentary screen-based behaviours than children living in lower-risk home environments [11, 28]. Higher-risk home environments were also associated with higher BMI-SDS at age 12 but not age 4 [11, 28], suggesting that effects on weight may not manifest until later childhood. However, the cross-sectional nature of these studies prevents understanding of the directionality of associations.

To our knowledge, no previous studies have used comprehensive measures to capture the home environment at multiple time points, limiting understanding of how the home environment and relationships with child weight change over time, as children transition from early childhood into adolescence. The present study utilised a comprehensive measure of the home environment to explore: (1) how the obesogenic nature of the home environment tracks over time, and (2) bi-directional associations between the home environment and child BMI-SDS from ages 4 to 12.

## Methods

### Sample

Participants were part of the Gemini study, a longitudinal birth cohort of families with twins born in England and Wales between March and December 2007. Gemini was established to examine genetic and environmental influences on energy balance behaviours and weight development during childhood [29]. A total of 2402 families with monozygotic (identical) and dizygotic (non-identical) twins consented to take part and completed baseline questionnaires when their twins were on average 8.2 months old (SD = 2.2), additional details on recruitment, data collection and baseline characteristics are provided elsewhere [29]. The Gemini cohort is largely representative of the UK population for most baseline characteristics, except for maternal age and education [29]. As described elsewhere [11], families were invited to take part in a home environment interview (HEI) when the children were on average 4.2 years old (SD = 0.4). Families who participated in the HEI at age 4 (*n* = 1113 families, *n* = 2226 children) were invited to participate in the HEI again when the children were on average 12.51 years old (SD = 0.22) [28]. Only families who had taken part in the HEI at age 4 (*n* = 1113), and those who completed caregiver feeding practices questionnaires in the month prior (*n* = 219 families, 438 children), were invited to take part in the present study. Of those invited to take part, a total of 149 families (68.0%) completed the HEI at age 12.

The study sample (*n* = 149 families, *n* = 298 children) comprised families with data on all variables included in the analysis. Compared with the full sample of families that completed the HEI at age 4 (*n* = 1113), parents in the current sample were slightly older at the child’s birth (35.1 (4.23) vs 33.7 (4.79)), and were of higher SES (5.03 (1.01) vs 4.48 (1.28)). There were no differences in maternal BMI at baseline, gestational age, sex of the child, birth weight or BMI-SDS at 4 years. When comparing the current sample to those completing the HEI at age 4, no significant differences were observed between the food and activity composite scores, but differences were observed for the media composite and the overall home environment composite.

### Measures

#### Home Environment Interview

Primary caregivers completed the HEI by telephone with a trained researcher when their children were 4 years of age, and again when they were 12 years of age. The HEI is a comprehensive measure of the home environment which assesses a range of physical and social aspects of the home food, physical activity and media environments [11, 28]. Caregiver feeding practices were assessed using validated questionnaires [30–33]. The HEI was originally developed for use in families with pre-school-aged children [11], and later updated for school-aged children [28]. Full details of the development and adaptation of the HEI, which was informed by literature review, consultation with a panel of childhood obesity experts and piloting with parents, are described in detail elsewhere [11, 28]. As described elsewhere [11, 28], the obesogenic quality of the home environment was determined by creating composite scores. A total of 32 constructs were included in the composite scores. Constructs incorporated aspects such as access to garden space, caregiver modelling of energy balance behaviours, availability and access to foods and beverages in the home, amongst other things (see Supplementary Table 1 for full list of constructs). Constructs identified as being associated with decreased risk for excess childhood weight gain were reverse-scored so that a higher total score would reflect ‘higher-risk’ for excess weight gain. Each variable was standardised using *z*-scores for the total sample at age 4 and age 12 and the standardised variables were summed to create three composite scores: the home food environment (21 variables), the physical activity environment (6 variables) and the media environment (5 variables). The food, activity and media composites were then summed to create an overall home environment composite score, dividing by the number of variables per composite so that each composite contributed equally to the overall score (food composite/21 + activity composite/6 + media composite/5). Higher scores on each composite reflect ‘higher-risk’ environments [11, 28].

The HEI was shown to have acceptable to high test-retest reliability over a two-week period at 4 and 12 years. Intraclass correlation coefficient (ICC); 95% confidence interval (CI) at age 4 was: food (ICC; 95% CI = 0.71; 0.52–0.83), activity (0.83; 0.72–0.91), media (0.92; 0.85–0.95), overall (0.92; 0.86–0.96) [11] and at age 12 was: food (0.77; 0.52–0.90), activity (0.62; 0.27–0.83), media (0.83; 0.61–0.93), overall (0.76; 0.49–0.90) [28]. Additionally, at age 4 the HEI had good to excellent validity when compared with images from a wearable camera of the home environment [34].

#### Anthropometric measurement

Information on weight at birth was obtained from the child’s health record and reported by the primary caregivers. Electronic weighing scales and height charts were sent to all Gemini families when the children were two years old and updated height charts were sent when the children were 10 years old for parents to report measurements at three-month intervals. At the time of the HEI, parents were also asked to provide their child’s height and weight measurements. Age, sex and gestational age were parent reported. Standard deviation scores (SDS) for child BMI (BMI-SDS) were calculated using the UK90 British growth reference data [35], adjusting for age, sex, and gestational age. Maternal BMI at baseline and 12 years was calculated using self-reported height and weight squared (kg/m^2^).

### Covariates

The following covariates were included, as previous literature indicated that they may be related to the predictor and outcome variables: child age at measurement, sex of child, and baseline maternal BMI [36, 37]. Due to the homogenous nature of the sample, ethnicity was not included as a covariate. Socioeconomic status (SES) was not included as a covariate as it likely sits on a causal pathway with child weight status [38].

### Statistical analysis

Pearson’s correlation was used to assess the continuity of the home environment composites from ages 4 to 12 years. Partial correlations were also calculated controlling for the time interval between each HEI. Paired samples t-tests were employed to examine differences in the home environment composite scores (overall and for the food, activity and media domains) between 4 and 12 years old. Again, the analysis was re-run controlling for the time interval between each HEI. There were no differences in mean HE composite score change by sex or zygosity so data from the whole sample were used in the final analyses. To understand how the home environment changed from ages 4 to 12, paired samples t-tests or McNemar’s tests were used to examine differences in the raw scores for the constructs included in the home environment composite scores (food, activity and media domains). These analyses were conducted using SPSS v26, with an alpha level of 0.05.

A cross-lagged structural equation model (SEM) was used to estimate effects of the obesogenic quality of the home environment on child BMI-SDS and vice versa. These analyses examined cross-sectional correlations between the overall home environment composite and BMI-SDS at ages 4 and 12, as well as prospective associations between the home environment composites and BMI-SDS at both time points. This process was repeated for the separate food, activity, and media composites. Age at measurement, sex of child, and maternal BMI were entered as covariates. All analyses were conducted in R version 4.0.3 using the statistical package lavaan [39] and the add-on lavaan.survey [40] which allows adjustment for clustering of twins within families. Utilising this approach means that both twins in a pair can be included in the analyses, maximising the sample size and statistical power. Standardised *β* were used to determine and compare the strength of associations. Model fit indices were calculated, with cut-offs in parentheses indicating acceptable to good fit: Comparative Fit Index (CFI ≥ 0.95), Tucker–Lewis Index (TLI; acceptable ≥0.90; good ≥0.95), Root Mean Square Error of Approximation (RMSEA ≤ 0.06), Standardised Root Mean Square Residuals (SRMR ≤ 0.08), chi-square (*χ* *p* > 0.05) [41]. The CFI, TLI, RMSEA, SRMR and variance explained (*R*^2^) are presented for each model [42]. Again, the analyses were re-run controlling for the time interval between each HEI. Previous literature suggests that any SEM model should include more than 200 participants [43], the current study included 298 participants at both time points.

## Results

### Sample characteristics

The analysis sample comprised 149 families (*n* = 298 children). All responders were the primary caregiver, with 98.7% (*n* = 147) being the child’s mother and 1.3% (*n* = 2) the father. Characteristics of the sample at ages 4 and 12 are outlined in Table 1.

**Table 1:** Characteristics of sample (*n* = 149 families, *n* = 298 children).

	Mean (SD) or % (*n*)
*Maternal characteristics*	
Maternal age at birth (years)	35.1 (4.23)
Maternal BMI at baseline	24.33 (4.19)
Maternal BMI at 12 year measurement	25.18 (4.79)
Maternal ethnicity	
White	94.6 (141)
Non-white	5.4 (8)
Marital status at baseline	
Married or cohabiting	98.7 (147)
Separated or divorced	0.7 (1)
Single	0.7 (1)
Marital status at 12 year measurement	
Married or cohabiting	94 (140)
Separated or divorced	4 (6)
Single	2 (3)
*Child characteristics *	
Sex of child (boys)	48.7 (145)
Zygosity^a^	
MZ pairs	28.9 (43)
DZ pairs	70.5 (105)
Gestational age (weeks)	36.07 (2.6)
Birth weight, SDS	−0.57 (0.96)
Age of child at 4 years measurement	4.08 (0.43)
Age of child at 12 years measurement	12.51 (0.22)
BMI-SDS of child at 4 years measurement	0.02 (0.87)
BMI-SDS of child at 12 years measurement	−0.06 (1.14)
*Home environment composites*	*Range*
Food environment at 4 years	−18.93 to 15.87
Physical activity environment at 4 years	−4.94 to 10.90
Media environment at 4 years	−6.45 to 18.11
Overall home environment at 4 years	−2.11 to 2.92
Food environment at 12 years	−13.67 to 23.15
Physical activity environment at 12 years	−4.54 to 15.45
Media environment at 12 years	−5.45 to 9.31
Overall home environment at 12 years	−2.17 to 3.02

^a^Zygosity information was missing for one family (*n* = 2 children).

#### Stability and continuity of home environment over time

Correlations over time for each of the home environment composites are presented in Table 2. The strength of associations were moderate to large for all correlations; ranging from 0.30 for activity composite and 0.64 for the media composite (*p* < 0.001). Partial correlations controlling for time difference between the visits produced almost identical results (not tabulated).

**Table 2:** Bivariate correlations for the home environment composite scores between age 4 and 12 years.

		Home environment at age 4				Home environment at age 12			
		Food composite	Activity composite	Media composite	Overall HE composite	Food composite	Activity composite	Media composite	Overall HE composite
Home environment at age 4	Food composite	1.00	0.23***	0.13*	0.57***	0.45***	0.14*	0.18***	0.34***
	Activity composite		1.00	−0.070	0.65***	0.13*	0.30***	0.02	0.21***
	Media composite			1.00	0.64***	0.32***	0.12*	0.64***	0.57***
	Overall HE composite				1.00	0.44***	0.31***	0.48***	0.60***
Home environment at age 12	Food composite					1.00	0.21*	0.37***	0.67***
	Activity composite						1.00	0.03	0.60***
	Media composite							1.00	0.76***
	Overall HE composite								1.00

*p < 0.05, **p < 0.01, ***p < 0.001.

Paired Samples *t*-tests assessed the stability of the home environment composites between ages 4 and 12, revealing the food (*t* = −2.37, *p* = 0.018), media (*t* = −7.22, *p* < 0.001) and overall HE (*t* = −4.63, *p* < 0.001) composite scores were higher (more obesogenic) at age 12 than age 4. No differences were observed for the activity composite (*t* = 0.52, *p* = 0.606) between 4 and 12 years. Paired Samples *t*-tests or McNemar’s tests were used to compare raw scores for individual constructs included in the home environment composite scores between ages 4 and 12. The mean number of energy-dense snacks available in the home increased between ages 4 (4.97 ± 2.14) and 12 (6.96 ± 3.22; *p* < 0.001). Similar findings were observed for the availability of sugar-sweetened beverages in the home (age 4 = 0.51 ± 0.78; age 12 = 1.44 ± 1.05; *p* < 0.001), and the number of electronic media devices present in the home (age 4 = 4.98 ± 2.30; age 12 = 15.48 ± 4.20; *p* < 0.001) and in children’s bedrooms (age 4 = 0.07 ± 0.29; age 12 = 1.70 ± 1.37; *p* < 0.001), indicating that these aspects of the home environment became more obesogenic from 4 to 12 years. Raw scores for the home environment constructs included in the composite scores at age 4 and age 12 are shown in Supplementary Table 2.

#### Bi-directional associations between HE and BMI-SDS

**Fig. 1:**
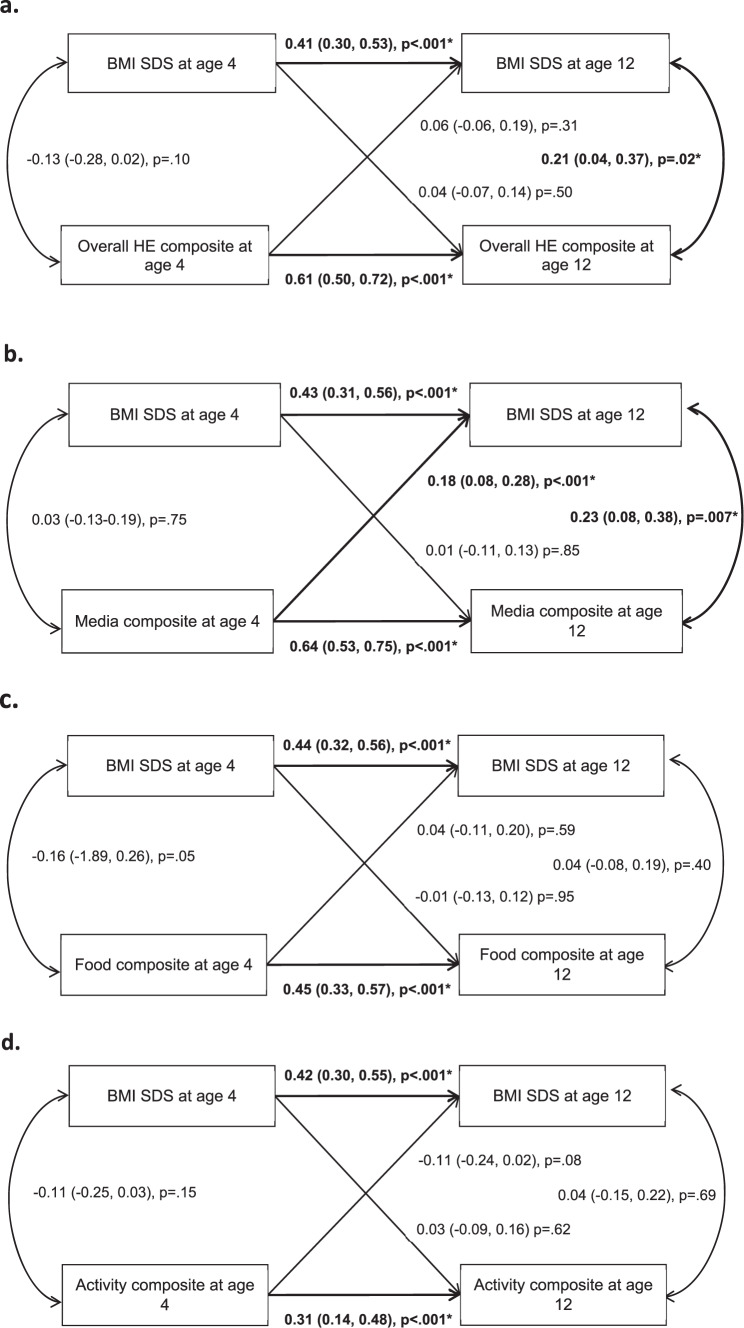
Cross-lagged models showing the associations between the home environment composites and BMI-SDS at ages 4 and 12 and vice versa. **a** Cross-lagged model showing the associations between the overall home environment composite and BMI-SDS at ages 4 and 12 and vice versa. Analyses were adjusted for clustering within families and covariates; age of child at measurement, sex of child, and maternal BMI at baseline. *denotes statistical significance. CFI: 0.98; TLI: 0.94; RMSEA: 0.04 (*p* = 0.57); SRMR: 0.05. *R*^2^ HE at age 12 = 0.37, *R*^2^ BMI-SDS age 12: 0.17. **b** Cross-lagged model showing the associations between the home media environment composite and BMI-SDS at ages 4 and 12 and vice versa. Analyses were adjusted for clustering within families and covariates; age of child at measurement, sex of child, and maternal BMI. CFI: 0.98; TLI: 0.96; RMSEA: 0.03 (*p* = 0.75); SRMR: 0.05. R^2^ home media composite at age 12 = 0.41, *R*^2^ BMI-SDS age 12: 0.22. **c** Cross-lagged model showing the associations between the home food environment composite and BMI-SDS at ages 4 and 12 and vice versa. Analyses were adjusted for clustering within families and covariates; age of child at measurement, sex of child, and maternal BMI. CFI: 0.98; TLI: 0.94; RMSEA: 0.03 (*p* = 0.75); SRMR: 0.05. *R*^2^ home food composite at age 12 = 0.20, *R*^2^ BMI-SDS age 12: 0.19. **d** Cross-lagged model showing the associations between the home activity environment composite and BMI-SDS at ages 4 and 12 and vice versa. Analyses were adjusted for clustering within families and covariates; age of child at measurement, sex of child, and maternal BMI. *denotes statistical significance CFI: 0.97; TLI: 0.94; RMSEA: 0.03 (*p* = 0.78); SRMR: 0.05. *R*^2^ home activity composite at age 12 = 0.09, *R*^2^ BMI-SDS age 12: 0.20.

Cross-lagged analyses for the overall HE composite and BMI-SDS at ages 4 and 12 and vice versa are shown in Fig. 1a. The findings revealed children’s BMI-SDS tracked from ages 4 to 12 *(β* = 0.41; 0.30, 0.53, *p* < 0.001) and the home environment tracked strongly from ages 4 to 12 (*β* = 0.61; 0.50, 0.72, *p* < 0.001). Analyses revealed a small positive cross-sectional correlation between the home environment and BMI-SDS at age 12 *(r* = 0.21, *p* = 0.02). The cross-lagged paths were not significant in either direction, indicating the home environment did not predict longitudinal changes in BMI-SDS, nor did BMI-SDS at age 4 predict longitudinal changes in the home environment to age 12.

Cross-sectional and cross-lagged analyses between the home media environment and BMI-SDS are shown in Fig. 1b. The findings revealed the home media environment tracked strongly from ages 4 to 12 (*β* = 0.64; 0.53, 0.75, *p* < .001). Analyses indicated a small positive cross-sectional correlation between the home media environment and BMI-SDS at age 12 *(r* = 0.23, *p* < 0.05). The cross-lagged paths revealed a significant but small relationship between the media composite at age 4 and BMI-SDS at age 12 *(β* = 0.18; 0.08, 0.28, *p* < 0.001), indicating that a ‘higher-risk’ media environment at age 4 predicted higher BMI-SDS at age 12. However, the reverse relationship from BMI-SDS at age 4 to media composite at age 12 was not significant.

Cross-lagged analyses for the home food and the home physical activity composites are presented in Fig. 1c, d, respectively. Pathways were not significant in either direction.

Sensitivity analyses controlling for time interval differences between each HEI produced almost identical results to those presented above (not reported here for brevity).

## Discussion

To our knowledge, this is the first study to utilise a comprehensive measure of the home environment to examine longitudinally the stability and continuity of the obesogenic home environment across childhood, and the first to examine bi-directional relationships between the obesogenic nature of the home environment and child BMI-SDS. Cross-sectional associations were observed between both the overall and media domains of the home environment and child BMI-SDS at 12 years, but not 4 years. Cross-lagged paths also revealed that the media composite score at age 4 was positively associated with child BMI-SDS at age 12, suggesting that living in a more obesogenic media environment predicted greater increases in child BMI-SDS from ages 4 to 12 years. There were no associations for the reverse paths, from BMI-SDS at age 4 to media composite at age 12, nor were associations observed between the other home environment composite scores (the overall, the food and activity composites respectively) and BMI-SDS.

The home environment showed moderate to strong tracking from ages of 4 to 12 years, with correlations ranging from 0.30 to 0.64 for all home environment composites (the overall HE composite, as well as food, activity and media domains) across the two time points. These findings indicate that children living in higher-risk, more obesogenic, home environments at age 4 tended to remain in higher-risk environments at age 12 and, similarly, those in lower-risk home environments at age 4 tended to remain in lower-risk environments at age 12. While findings indicated continuity in the home environment composites at an individual level, significant increases were observed for the food, media and overall home environment composite scores from ages 4 to 12. Significant differences were also observed for the individual constructs that comprise the composite scores, with notable increases in the availability of energy-dense snacks and sugar-sweetened beverages in the home, as well as significant increases in electronic devices both in the home and children’s bedrooms between ages 4 and 12. Together these findings indicate that while each household tends to keep their relative position in the obesogenic nature of their home environment, aspects of the home environments became more obesogenic in nature over the 8-year period. The increases in availability of electronic devices may also in part reflect societal and technological developments over the past decade [44]. Age-related increases in the obesogenic nature of the home environment have been demonstrated previously, with reported decreases in the frequency of family mealtimes as children get older [45], and increases in availability and access to electronic devices as children reach adolescence [44]. The tracking of the home environment over time highlights the importance of early intervention to try to support families to establish a home environment that encourages healthy eating, physical activity and age-appropriate media use, from early childhood.

In accordance with previous research [8], the most consistent relationships between the home environment and child BMI-SDS were observed in the media domain, with the media composite at age 4 predicting changes in child BMI-SDS from age 4 to 12. In addition, cross-sectional associations were observed between child BMI-SDS and the home media environment. These findings suggest that the media environment that a child is exposed to in early life (age 4) predicts greater increases in BMI-SDS from age 4 to 12, and that a child’s BMI-SDS continues to be influenced by the media environment they are exposed to in later childhood (age 12). It is however important to note that the cross-sectional nature of the association at age 12 means we are unable to determine directionality, and thus it may be that a child’s BMI-SDS at age 12 influences the media environment or that the two influence each other. These findings suggest that relationships between certain aspects of the home environment and child weight may not manifest until later childhood. One possible reason for this may be that older children have more autonomy over their food choices and behaviours than younger children and are also exposed to a wider range of external obesogenic influences, which may have a cumulative effect on weight [46]. Our findings build on previous longitudinal research highlighting potential relationships between individual aspects of the media environment and child adiposity [8]. The longitudinal relationship between the home media environment at age 4 and child BMI-SDS eight years later, suggests the media environment plays an influential role in shaping children’s weight trajectories, and thus may be an important avenue to explore when designing obesity prevention and treatment strategies.

There are a number of potential explanations as to why the media environment has been found to associated with child weight. Firstly, the media environment within the family home is correlated with activity levels and screen-based sedentary behaviours that have been associated with risk of excess weight gain [28]. Secondly, the observed relationships between the home media environment and child weight may partly reflect the fact that the media domain is more stable, less complex, and therefore easier to characterise and measure than other aspects of the home environment [8]. Finally, the media environment may be less susceptible to social desirability biases and therefore more accurately reported, when compared to the food or physical activity domains. Such biases may vary dependent on child weight status [47], with parents of children with overweight or obesity more susceptible to desirability bias, which can make it difficult to disentangle the role of the home environment in child weight development.

Pathways between the overall home environment composite and child BMI-SDS were not significant in either direction. Similar results were observed for the home food and activity composites. These findings may partly result from the small size and limited diversity (in terms of ethnicity and socioeconomic status) of the present sample. Children of lower socioeconomic status (SES) are more likely to live in more obesogenic home environments [12, 48–50], and have higher rates of adiposity than those of higher SES [51, 52]. In addition, there are difficulties in measuring some aspects of the home environment as they rely on parent-report, which is susceptible to biases. As such the true range of potential scores for the obesogenic home environment may not have been captured in the current sample, limiting the ability to uncover both cross-sectional and longitudinal relationships with BMI-SDS. Future research should replicate the findings in a more generalisable sample.

Another potential explanation for the lack of association between the overall home environment composite and child BMI-SDS could be that these relationships are complex, involving gene-environment interactions. Individual variation in susceptibility to obesogenic environments may influence associations between the home environment and child weight trajectories [53]. In line with this, research has demonstrated that the heritability of BMI is significantly higher for children living in more obesogenic home environments compared to those from healthier homes (heritability of 86% vs 39%) [54]; suggesting children with greater genetic susceptibility to obesity are at greater risk of developing obesity when they grow up in more obesogenic environments [54].

The finding of no clear relationship between child BMI-SDS and either the food or physical activity domains reflect the inconsistency of previous evidence in this area [8]. Reviews have generally found an absence of convincing evidence for the contribution of physical activity to child adiposity [55, 56]. The home food environment is similarly complex [7], and is influenced by both social and physical factors [8, 11]. Previous research has shown clear associations between the home food environment and children’s food intake, with more ‘obesogenic’ home food environments associated with lower frequency of fruit and vegetable consumption and higher frequency of energy-dense snack consumption at both ages 4 and 12 [11]. However, the frequency with which different varieties of foods are consumed does not necessarily equate to overall energy intake or directly impact weight status. Furthermore, unlike the media and activity environment, the food environment is more likely to fluctuate from day-to-day and vary with seasonal changes, adding to the complexity of measurement.

### Strengths and limitations

The strengths of this study include the prospective study design, with repeated measures of the comprehensive obesogenic home environment and children’s heights and weights at ages 4 and 12, and the ability to control for important confounding variables. Despite this, there are a number of limitations to this study that need to be considered. The HEI is parent reported and thus susceptible to social desirability and recall biases [57, 58], and such biases may vary dependent on child weight status [47], which can make it difficult to disentangle the role of the home environment in child weight development and must be taken into consideration when interpreting the results. However, as mentioned in the methods, the HEI has shown acceptable to high test-retest reliability over a two-week period, and good to excellent validity when compared with images from a wearable camera of the home environment [34]. Furthermore, whilst the development of the HEI was guided by expert consultation, comprehensive review of the literature and piloting with the target population [8, 28], and the home environment composites incorporated many factors agreed to be relevant to risk for weight gain [28], it is possible that some relevant factors, such as sleep, were not included or captured adequately. Additionally, the sample size was small and relatively homogenous, with a large proportion of higher SES households and White-British compared to the general population, meaning findings may not be representative. Future research should aim to replicate our findings in a larger, more socioeconomically and ethnically diverse sample. The use of a larger sample size would be beneficial as it would increase the statistical power to detect small effects, it is possible that we were underpowered in this study. Furthermore, the majority of primary caregivers in this study were mothers (98.7%). Although primary caregivers were also asked to provide responses for their partner, it is possible that responses provided by the mothers may differ from views of the fathers/co-parent, as has been found in previous research examining differences between maternal and paternal roles and perceptions with respect to feeding their children [59]. It is, therefore, important for future research to gain greater representation from fathers and co-parents, given that they directly and indirectly contribute to the home environment, and thereby shape risk of child obesity. Thirdly, height and weight measurements were parent reported, which may introduce inaccuracies and bias, however, parent reports have been shown to correspond with researcher-report [60] and more so when parents are provided with electronic scales and height charts [61], as was the case in this study. Finally, the use of BMI-SDS as the primary measure of adiposity is a limitation, as it cannot differentiate between weight attributable to fat mass or lean mass therefore misclassification of weight status can occur at an individual level, especially during childhood when maturation occurs at differing rates. Thus, utilising other measures of adiposity such as waist circumference, body fat percentage, or skinfold thickness may be beneficial.

## Conclusion

This is the first study to explore how physical and social aspects of the food, activity and media environments of the family home change from early childhood into adolescence. This study provides evidence for the tracking of the obesogenic home environment across childhood and, in particular, highlights the importance of the early home media environment for child weight development. These findings provide important insight into key aspects of the home environment, such as the media environment, that could be targeted when developing obesity treatment or prevention strategies.

## Supplementary information

Supplementary Tables 1 and 2

## Data Availability

The data that support the findings of the current study are available from the corresponding author on reasonable request, following approval from the Gemini study.
