# Supplementary material for: Associations between the home environment and childhood weight change: a cross-lagged panel analysis
Source: Int J Obes (Lond). 2022 Jun 24;46(9):1678–85. doi: 10.1038/s41366-022-01170-8 (PMC9395269; doi:10.1038/s41366-022-01170-8)
Supplement: Supplementary file 1 — Supplementary Tables 1 and 2 [file 41366_2022_1170_MOESM1_ESM.docx]

**Supplementary Materials**

**Supplementary Table 1.** Constructs included in the home environment composite score. (Items coloured red are those added to the original composite score during the update).

**Supplementary Table 2.** Means (±SD) or % (N) for the home environment constructs included in the composite scores at age 4 and age 12 and significance of differences between time points (Paired samples t-test or McNemar’s test).

**Supplementary Table 1.** Constructs included in the home environment composite score. (Items coloured red are those added to the original composite score during the update).

| **Version 1: HE Composite 2012** | **Version 2: Updated HE Composite** |
| --- | --- |
| **Food-related constructs (21)** | **Food-related constructs (21)** |
| ***Availability*** | ***Availability*** |
| Number of fruit types[^1^](https://www.sciencedirect.com/science/article/pii/S0195666316304251#tbl1fna) | Number of fruit types[^1^](https://www.sciencedirect.com/science/article/pii/S0195666316304251#tbl1fna) |
| Number of vegetable types[^1^](https://www.sciencedirect.com/science/article/pii/S0195666316304251#tbl1fna) | Number of vegetable types[^1^](https://www.sciencedirect.com/science/article/pii/S0195666316304251#tbl1fna) |
| Number of energy-dense snack types | Number of energy-dense snack types |
| Number of sugar-sweetened drinks | Number of sugar-sweetened drinks |
| ***Accessibility (visibility)*** | ***Accessibility (visibility)*** |
| Fruit on display[^1^](https://www.sciencedirect.com/science/article/pii/S0195666316304251#tbl1fna) | Fruit on display[^1^](https://www.sciencedirect.com/science/article/pii/S0195666316304251#tbl1fna) |
| Vegetables ready-to-eat[^1^](https://www.sciencedirect.com/science/article/pii/S0195666316304251#tbl1fna) | Vegetables ready-to-eat[^1^](https://www.sciencedirect.com/science/article/pii/S0195666316304251#tbl1fna) |
| Energy-dense snacks on display | Energy-dense snacks on display |
| Sugar-sweetened drinks on display | Sugar-sweetened drinks on display |
| ***Accessibility (child can help him/herself)*** | ***Accessibility (child can help him/herself)*** |
| Fruit[^1^](https://www.sciencedirect.com/science/article/pii/S0195666316304251#tbl1fna) | Fruit[^1^](https://www.sciencedirect.com/science/article/pii/S0195666316304251#tbl1fna) |
| Vegetables[^1^](https://www.sciencedirect.com/science/article/pii/S0195666316304251#tbl1fna) | Vegetables[^1^](https://www.sciencedirect.com/science/article/pii/S0195666316304251#tbl1fna) |
| Energy-dense snacks | Energy-dense snacks |
| Sugar-sweetened drinks | Sugar-sweetened drinks |
| ***Parental feeding practices*** | ***Parental feeding practices*** |
| Emotional feeding | Emotional feeding |
| Instrumental feeding | Instrumental feeding |
| Encouragement[^1^](https://www.sciencedirect.com/science/article/pii/S0195666316304251#tbl1fna) | Encouragement[^1^](https://www.sciencedirect.com/science/article/pii/S0195666316304251#tbl1fna) |
| Modelling[^1^](https://www.sciencedirect.com/science/article/pii/S0195666316304251#tbl1fna) | Modelling[^1^](https://www.sciencedirect.com/science/article/pii/S0195666316304251#tbl1fna) |
| Monitoring[^1^](https://www.sciencedirect.com/science/article/pii/S0195666316304251#tbl1fna) | Monitoring[^1^](https://www.sciencedirect.com/science/article/pii/S0195666316304251#tbl1fna) |
| Covert restriction[^1^](https://www.sciencedirect.com/science/article/pii/S0195666316304251#tbl1fna) | Covert restriction[^1^](https://www.sciencedirect.com/science/article/pii/S0195666316304251#tbl1fna) |
| Restriction[^1^](https://www.sciencedirect.com/science/article/pii/S0195666316304251#tbl1fna) | Restriction[^1^](https://www.sciencedirect.com/science/article/pii/S0195666316304251#tbl1fna) |
| Family meal frequency at the table | Family meal frequency at the table |
| Frequency child eats while watching TV | Frequency child eats while watching TV and/or using a device |
| **Physical activity-related constructs (6)** | **Physical activity-related constructs (6)** |
| Garden/outdoor space[^1^](https://www.sciencedirect.com/science/article/pii/S0195666316304251#tbl1fna) | Garden/outdoor space[^1^](https://www.sciencedirect.com/science/article/pii/S0195666316304251#tbl1fna) |
| Garden play equipment[^1^](https://www.sciencedirect.com/science/article/pii/S0195666316304251#tbl1fna) | Garden play equipment[^1^](https://www.sciencedirect.com/science/article/pii/S0195666316304251#tbl1fna) |
| Allowed to play indoors[^1^](https://www.sciencedirect.com/science/article/pii/S0195666316304251#tbl1fna) | Allowed to play indoors[^1^](https://www.sciencedirect.com/science/article/pii/S0195666316304251#tbl1fna) |
| Allowed to play outdoors[^1^](https://www.sciencedirect.com/science/article/pii/S0195666316304251#tbl1fna) | Allowed to play outdoors[^1^](https://www.sciencedirect.com/science/article/pii/S0195666316304251#tbl1fna) |
| Caregiver modelling of physical activity[^1^](https://www.sciencedirect.com/science/article/pii/S0195666316304251#tbl1fna) | Caregiver modelling of physical activity[^1^](https://www.sciencedirect.com/science/article/pii/S0195666316304251#tbl1fna) |
| Caregiver support of physical activity[^1^](https://www.sciencedirect.com/science/article/pii/S0195666316304251#tbl1fna) | Caregiver support of physical activity[^1^](https://www.sciencedirect.com/science/article/pii/S0195666316304251#tbl1fna) |
| **Media-related constructs (5)** | **Media-related constructs (5)** |
| Number of media equipment in home | Number of media equipment in home |
| TV in the child’s bedroom | Greater number of media equipment in child’s bedroom |
| Household rules around media use[^1^](https://www.sciencedirect.com/science/article/pii/S0195666316304251#tbl1fna) | Caregiver rules around use of media equipment[^1^](https://www.sciencedirect.com/science/article/pii/S0195666316304251#tbl1fna) |
| TV viewing of primary caregiver (hrs. per week) | Primary caregiver time engaged in screen-based viewing (hrs. per week) |
| TV viewing of partner (hrs. per week) | Partner time engaged in screen-based viewing (hrs. per week) |
| ^1^ Variable was identified as being associated with decreased risk for weight gain. | |

**Supplementary Table 2.** Means (±SD) or % (N) for the home environment constructs included in the composite scores at age 4 and age 12 and significance of differences between time points (Paired samples t-test or McNemar’s test).

| **Home food environment** | **Age 4** | **Age 12** | **Significance of 4-12 years difference** |
| --- | --- | --- | --- |
| ***Availability*** |  |  |  |
| Number of fruit types[^1^](https://www.sciencedirect.com/science/article/pii/S0195666316304251#tbl1fna) | 8.48 (2.98) | 9.65 (4.25) | t=-5.57, p<0.001 |
| Number of vegetable types[^1^](https://www.sciencedirect.com/science/article/pii/S0195666316304251#tbl1fna) | 11.57 (4.11) | 13.58 (4.63) | t=-7.38, p<0.001 |
| Number of energy-dense snack types | 4.97 (2.14) | 6.96 (3.22) | t=-10.49, p<0.001 |
| Number of sugar-sweetened drink types | 0.51 (0.78) | 1.44 (1.05) | t=-14.04, p<0.001 |
| ***Accessibility (visibility)*** |  |  |  |
| Fruit on display[^1^](https://www.sciencedirect.com/science/article/pii/S0195666316304251#tbl1fna) | 94.6 (282) | 95.3 (284) | ꭕ^2^=3.29, p=0.070 |
| Vegetables ready-to-eat[^1^](https://www.sciencedirect.com/science/article/pii/S0195666316304251#tbl1fna) | 50.3 (150) | 43 (128) | ꭕ^2^=0.04, p=0.845 |
| Energy-dense snacks on display | 15.4 (46) | 4.0 (12) | ꭕ^2^=21.78, p<0.001 |
| Sugar-sweetened drinks on display | 8.7 (26) | 6.0 (18) | ꭕ^2^=1.75, p=0.186 |
| ***Accessibility (child can help him/herself)*** |  |  |  |
| Fruit[^1^](https://www.sciencedirect.com/science/article/pii/S0195666316304251#tbl1fna) | 77.9 (232) | 92.6 (276) | ꭕ^2^=214.00, p<0.001 |
| Vegetables[^1^](https://www.sciencedirect.com/science/article/pii/S0195666316304251#tbl1fna) | 51.7 (154) | 94.6 (282) | ꭕ^2^=109.26, p<0.001 |
| Energy-dense snacks | 8.1 (24) | 55.4 (165) | ꭕ^2^=131.54, p<0.001 |
| Sugar-sweetened drinks | 2.7 (8) | 41.6 (124) | ꭕ^2^=103.32, p<0.001 |
| ***Parental feeding practices*** |  |  |  |
| Emotional feeding^2^ | 1.68 (0.55) | 1.45 (0.47) | t=7.64, p<0.001 |
| Instrumental feeding^2^ | 2.32 (0.64) | 1.81 (0.53) | t=15.52, p<0.001 |
| Encouragement[^1^](https://www.sciencedirect.com/science/article/pii/S0195666316304251#tbl1fna)^, 2^ | 1.93 (0.52) | 2.28 (0.59) | t=-11.43, p<0.001 |
| Modelling[^1^](https://www.sciencedirect.com/science/article/pii/S0195666316304251#tbl1fna)^,2^ | 2.29 (0.64) | 3.65 (0.68) | t=-21.45, p<0.001 |
| Monitoring[^1^](https://www.sciencedirect.com/science/article/pii/S0195666316304251#tbl1fna)^,2^ | 2.32 (0.93) | 2.44 (0.98) | t=-2.26, p=0.025 |
| Covert restriction[^1^](https://www.sciencedirect.com/science/article/pii/S0195666316304251#tbl1fna)^, 2^ | 2.90 (0.84) | 3.23 (0.89) | t=-6.68, p<0.001 |
| Restriction[^1^](https://www.sciencedirect.com/science/article/pii/S0195666316304251#tbl1fna)^, 3^ | 2.71 (1.07) | 3.52 (1.16) | t=-11.70, p<0.001 |
| Family meal frequency at the table (days per /week) | 4.00 (1.60) | 3.43 (1.22) | t=5.40, p<0.01 |
| Frequency child eats while watching TV and/or using a device (days per /week) | 1.03 (1.38) | 1.24 (1.19) | t=-2.52, p=0.012 |
| **Home activity environment** |  |  |  |
| Garden/outdoor space[^1^](https://www.sciencedirect.com/science/article/pii/S0195666316304251#tbl1fna) | 99.3 (296) | 98.7 (294) | X^2^ = NA^7^, p=0.687 |
| Garden play equipment[^1,^](https://www.sciencedirect.com/science/article/pii/S0195666316304251#tbl1fna) ^4^ | 81.9 (244) | 65.8 (196) | ꭕ^2^=23.01, p<0.001 |
| Allowed to be physically active indoors[^1^](https://www.sciencedirect.com/science/article/pii/S0195666316304251#tbl1fna)^, 4, 5^ | 4.70 (0.70) | 4.30 (1.07) | t=6.86, p<0.001 |
| Allowed to be physically active outdoors[^1^](https://www.sciencedirect.com/science/article/pii/S0195666316304251#tbl1fna)^, 4, 5^ | 4.31 (0.83) | 4.76 (0.56) | t=-5.82, p<0.001 |
| Parental modelling of physical activity[^1^](https://www.sciencedirect.com/science/article/pii/S0195666316304251#tbl1fna) | 3.95 (0.77) | 3.97 (0.96) | t=-0.24, p=0.813 |
| Parental support of physical activity[^1^](https://www.sciencedirect.com/science/article/pii/S0195666316304251#tbl1fna) | 4.04 (0.59) | 3.53 (0.77) | t=-13.75, p<0.001 |
| **Home media environment** |  |  |  |
| Number of media equipment items in home | 4.98 (2.30) | 15.48 (4.20) | t=-50.30, p<0.001 |
| Number of media equipment in child’s bedroom | 0.07 (0.29) | 1.70 (1.37) | t=-21.13, p<0.001 |
| Caregiver rules around use of media equipment[^1^](https://www.sciencedirect.com/science/article/pii/S0195666316304251#tbl1fna)^,6^ | 0.71 (0.45) | 2.38 (0.78) | t=-33.50, p<0.001 |
| Maternal time engaged in screen-based viewing (hours/week) | 14.73 (8.78) | 14.26 (8.55) | t=1.00, p=0.319 |
| Partner time engaged in screen-based viewing (hours/week) | 15.24 (9.53) | 14.94 (9.61) | t=0.43, p=0.667 |
| ^1^Variables identified as being associated with decreased risk for weight gain were reverse scored. However, in this table the scores are not reversed for comparison purposes. The only scores presented as reversed in this table are the Parental feeding practices: Encouragement, Modelling, Monitoring, Covert restriction and Restriction.  ^2^ Measured using a five-point Likert scale (1= never, 5= always).  ^3^ Measured using a seven-point Likert scale (1= not at all, 7= strictly).  ^4^n= 294 as four children did not have access to a garden or outdoor space.  ^5^Measured using a five-point Likert scale (1=never; 5=all the time)  ^6^(0 = no rules, 1=rules around one device, 2 = rules around two devices, 3 = rules around 3 or more devices)  ^7^ The number of discordant pairs was <25 therefore no chi-square value is provided and the exact test is used to represent the significance. | | | |
